# Colored Shade Nets and LED Lights at Different Wavelengths Increase the Production and Quality of Canada Goldenrod (*Solidago canadensis* L.) Flower Stems

**DOI:** 10.3390/plants14203119

**Published:** 2025-10-10

**Authors:** Fabíola Villa, Luciana Sabini da Silva Murara, Giordana Menegazzo da Silva, Edvan Costa da Silva, Larissa Hiromi Kiahara Sackser, Laís Romero Paula, Mateus Lopes Borduqui Cavalcante, Daniel Fernandes da Silva

**Affiliations:** 1 Post-Graduation Program in Agronomy, Western Parana State University (Unioeste), Rua Pernambuco, 1777, Centro, P.O. Box 91, Marechal Cândido Rondon 85960-128, PR, Brazil; fvilla2003@hotmail.com (F.V.); edvan_costa@outlook.com (E.C.d.S.); larissakiahara@hotmail.com (L.H.K.S.); laisromeropaula@gmail.com (L.R.P.); mateusborduqui@hotmail.com (M.L.B.C.); 2 Agronomy Course, Universidade Paranaense (Unipar), Unidade de Toledo (Campus I), Avenida Parigot de Souza, 3636, Jardim Prada, Toledo 85903-170, PR, Brazil; luciana.sabini@hotmail.com; 3 Post-Graduation Program in Plant Production, Departament of Botany, Federal University of Paraná (UFPR), Avenida Coronel Francisco Heráclito dos Santos, 100, Jardim das Américas, P.O. Box 19031, Curitiba 81531-980, PR, Brazil; giomenesilva_@hotmail.com

**Keywords:** *Solidago canadensis* L., cut flower, light quality, light spectrum, photoperiod

## Abstract

Canada goldenrod (*Solidago canadensis* L.), a short-day plant commonly cultivated as a cut flower, depends on proper lighting management to obtain long stems and higher commercial value. Thus, this study aimed to determine the effect of modifying the light spectrum through the installation of light-emitting diodes (LEDs) and the use of colored shade nets on the production and quality of Canada goldenrod stems. The treatments used were colored shade nets and different LED lighting treatments. Production per plant and productivity per square meter were determined. Twenty stems were selected and evaluated for: stem length; inflorescence length and width; number of floral ramets per inflorescence; number of leaves; stem base diameter (mm); and fresh stem biomass (g). Canada goldenrod plants require an extension of the light period with artificial lighting to produce higher-quality stems, regardless of whether the bulbs emit red or white light. The use of nets with 50% red and white shading promoted higher production and elongation of Canada goldenrod stems, with a production that reached up to 4.2 floral stems per plant and 100.3 floral stems per square meter using the red shade net and white LED. These floral stems were of high commercial standard, with a length of up to 81.35 cm with the red shade net and red LED, and were 31 cm in diameter for the inflorescences, approximately, under black or white shade nets and white or red LEDs. More robust floral stems with greater biomass were observed using any shade net color and LED lamps.

## 1. Introduction

Growth and development throughout the life cycle of plants are controlled by photoreceptors that act as timers for the main developmental transitions such as germination and the beginning of flowering [1,2]. Plants react differently to different light characteristics, such as spectral composition, intensity, duration, and direction, which influence their growth and development [3,4,5].

Light quality promotes important effects on the morphogenesis, photosynthesis, and flowering of plants [6,7]. The modulation of the incident spectrum becomes a tool capable of increasing gains in crop productivity and quality [8].

There are two ways of manipulating light quality in agriculture. The first consists of artificial lighting using lamps capable of emitting light at different wavelengths and specific spectral ranges, especially those of light-emitting diodes (LEDs) [9,10]. For example, Zheng and Labeke [11] reported on the importance of different wavelengths on the quality of chrysanthemum (*Chrysanthemum* × morifolium (Ramat.) Hemsl.), emphasizing that changes in the intensity and quality of light caused anatomical changes in the amount of photosynthetic pigment and biomass accumulation.

The second possibility is the use of colored shade nets, which provide physical protection, changes in environmental conditions, increases in the relative proportion of scattered light, and the absorption of various spectral ranges [12,13]. Silva et al. [14] observed that the use of blue-and black-colored shade nets with 35–40% and 45–49% light retention, respectively, resulted in higher production of heliconia ‘Golden Torch’ (*Heliconia psittacorum* L.f.) per area, as a result of the higher number of flower stems per plant and the higher number of stems within the commercialization standards for the species.

The development of all plants is linked to the light conditions to which they are subjected, especially those considered short-day plants such as solidago (*Solidago canadensis* L.) [15]. This species, native to North America, belongs to the Asteraceae family and is cultivated for ornamental purposes, as a cut flower, mainly intended for bouquet composition and floral arrangements, due to its large, erect, branched terminal inflorescences and numerous small capitula, which are yellowish green in color [16,17].

The use of light manipulation in the production of Solidago canadensis flower stems is a current reality. Castillo et al. (2025), comparing the use of intense LED lighting (1.64 µmol m^−2^ s^−1^) and diffuse lighting (0.21 µmol m^−2^ s^−1^), observed that under diffuse LED lighting, a lower percentage of plants entered early flowering and had a greater number of stems and a smaller stem diameter, although this did not influence the number of days to harvest [18].

The morphometric parameters of flower stems are generally the most commonly considered parameters for determining product quality. Because Canada goldenrod is a plant, differences occur among the stems produced, mainly in response to environmental variables and agronomic practices, with higher-quality stems being marketed with a higher added value. These differences in measurements allow for Canada goldenrod stems to be divided into classes, which can vary according to a combination of parameters, notably, stem length, which ranges from 40 to 80 cm, and weight, with a minimum acceptable weight for the Brazilian market of 10 g. Furthermore, the stem flowers must be straight and not present curvatures or inclinations or phytosanitary problems [19].

Although tango is widely cultivated, its production still encounters many obstacles of a phytotechnical nature, such as the adjustment of artificial lighting, which allows for higher-quality stems to be obtained and allows for the continuous production of flowers throughout the year, improving the agronomic profitability of the crop. In view of the above, the present study aimed to determine the effect of modifying the light spectrum through the installation of artificial lighting and the use of colored shade nets on the production and quality of tango stems.

## 2. Results

The data analysis showed a significant interaction between colored shade nets and the use of LED lights for the photoperiod extension, stem length, inflorescence width, number of stems per plant, and number of stems per square meter. Inflorescence length and fresh stem biomass showed significance for these factors alone, while the floral scape diameter showed a difference only for nets. Moreover, the number of leaves and number of floral branches showed no statistical difference.

**Figure 1:**
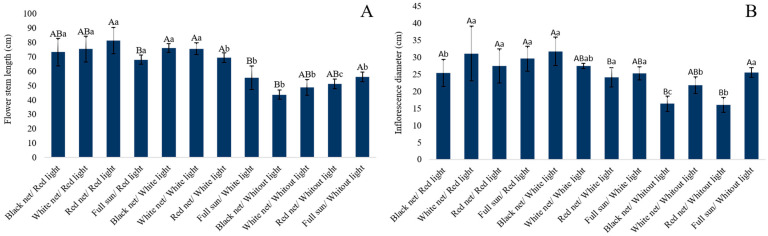
Stem length (**A**); and inflorescence diameter (**B**) of Canada goldenrod (*Solidago canadensis* L.), grown under light-emitting diodes (LEDs) with different light qualities and shade nets of different colors. Averages followed by the same uppercase letter differ from each other within the same LED illumination, and averages followed by the same lowercase letter differ from each other within the same-colored shade net, by Tukey’s test, at a 5% error probability.

The significant interaction between colored shade nets and the LED light is evident on plants with regard to stem elongation (Figure 1A), in which the use of artificial lighting increased the photoperiod, providing longer stems. Better results were obtained when using red light LEDs on plants grown under red-colored shade nets, followed by red and white light LEDs under white- and black-colored shade nets, which did not differ statistically from each other.

The inflorescence diameter data showed an interaction between colored shade nets and artificial lighting (Figure 1B). Canada goldenrod inflorescences under lighting conditions with red light LEDs were narrower only with plants grown under the black-colored shade net.

**Figure 2:**
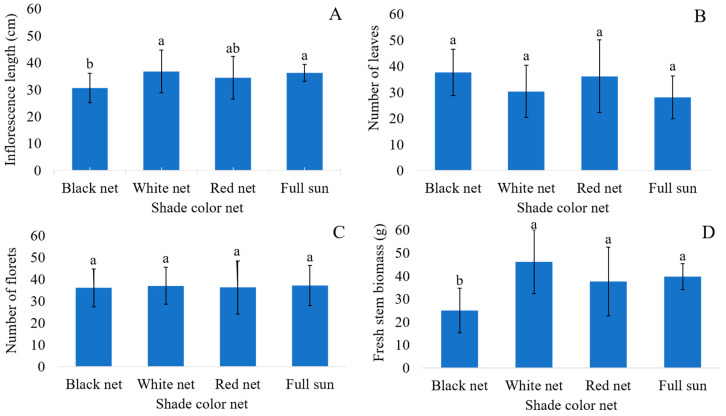
Inflorescence length (**A**); number of leaves (**B**); number of florets (**C**); and fresh stem biomass (**D**) of Canada goldenrod (*Solidago canadensis* L.) grown under shade nets of different colors. Averages with the same letter do not differ from each other within the shade net color according to Tukey’s test at a 5% error probability.

**Figure 3:**
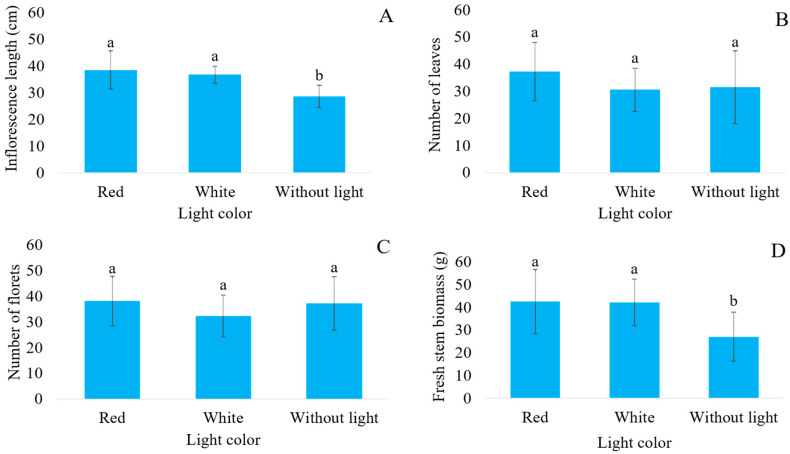
Inflorescence length (**A**); number of leaves (**B**); number of florets (**C**); and fresh stem biomass (**D**) of Canada goldenrod (*Solidago canadensis* L.) grown under LEDs with different light colors. Averages followed by the same letter do not differ from each other within the same LED illumination according to Tukey’s test at a 5% error probability.

The inflorescence length showed an isolated significance for the colored shade nets and the types of lighting (Figure 2A and Figure 3A). Inflorescences grown under the white-colored shade net and full sun had a higher length, which was not different than those grown under the red-colored shade net, which in turn did not differ from the length of the stems of plants grown under the black-colored shade net, although the latter was inferior to the first two (Figure 2A). Regarding the use of LED, greater inflorescence lengths were found in plants grown with the use of lighting, regardless of whether the LED light used was red or white (Figure 3A).

The number of leaves and florets showed no significant differences resulting from the different treatments (Figure 2B,C and Figure 3B,C). Changes promoted in the light spectrum both by colored shade nets and by LED lights did not cause photomorphogenic alterations that would increase the number of internodes and the differentiation of vegetative into reproductive buds in plants, with only an elongation of internodes.

Fresh stem biomass showed a significant difference in terms of the variables alone (Figure 2D and Figure 3D). Only the black-colored shade net was inferior, with a mean fresh stem biomass of 25.05 g, with no difference observed among the other colored shade nets (Figure 2D). Regarding lighting, stems grown under red or white LED lights did not differ from each other, but both were superior to the treatment without lighting (Figure 3D).

Regarding the inflorescence diameter, there was an interaction between the shade net color and artificial lighting (Figure 1A). Under lighting conditions with red LED light, the Canada goldenrod inflorescences were narrower, only when originating from plants grown under black-colored shade net. Stems grown under the same black-colored shade net with 50% shading, but with white light LEDs, presented the largest inflorescence diameter, with values of up to 31.71 cm, which did not differ statistically from treatments with white LED light under the white-colored shade net and full sun, but superior only to those observed using the red-colored shade net. The superior performance among the treatments that did not receive artificial lighting was verified by inflorescences produced in full sun despite not having exceeded 25.48 cm in width.

The base diameter of the flower scape showed a significant difference only for colored shade nets. Wider scapes were observed in plants grown without shading and with white and red-colored shade nets; however, the latter two did not differ statistically from those grown under the black-colored shade net, which presented narrower scapes measuring only 4.97 mm (Table 1).

The number of stems flower per plant presented a significant interaction between colored shade nets and LED light colors. The highest production was obtained with the use of the red-colored shade net and white LED light (4.2 stems per plant) (an increase of 110% compared to the control treatment without the use of a shading net and LED lighting), which showed a trend to increase despite not differing statistically from the white-colored shade net and the absence of a net. The use of red LED light showed no statistical difference between plants regarding the number of stems, regardless of the colored shade net, unlike treatments without LED light, in which colored shade nets influenced the emitted number of stems. The most notable was the white-colored shade net, which produced 3.2 stems per plant, which did not differ from the red-colored shade net and full sun conditions, which were all superior to plants grown under the black-colored shade net (Table 2).

Studying the different qualities of light offered by the LEDs in each shade net, the result for the number of stems per square meter was the same as that for the number of stems per plant; the only difference among the three lighting conditions was for the red shade net, in which productivity was higher when the white or red LEDs light were used in combination with the red-colored shade net.

As the number of stems per square meter is directly related to the number of plants per square meter and, consequently, the number of stems emitted per plant, the results for these variables were similar. Again, under red LED light there was no difference when associated with the covers. The best results—that is, the greater number of stems m^−2^—were found in plants grown under a red shade net intercropped with white light lamps (100.3 stems m^−2^), with an increase of 105% compared to the control treatment without the use of a shading net and LED lighting. There was no difference between plants grown with the white shade net and in full sun with the same type of LED light, although a tendency towards increased productivity under the red shade net can be observed (Table 2).

As can be seen by examining the number of stems per plant, the treatment combining the black shade net and no lighting proved to be inferior, differing from the productivity under the white shade net without lighting, but also similar to treatments the with red shade and full sun, which, in turn, were intermediaries.

## 3. Discussion

The stem lengths were longer with the use of artificial lighting with red and white LED lights combined with red- and white-colored shade nets. Red-colored shade nets transfer the light of the spectrum in the red and far-red wavelengths, with higher transmittances for wavelengths above 590 nm, reducing blue, green, and yellow wavelengths and acting in the development of the photosynthetic structure of plants. Therefore, some plant species may present starch accumulation by inhibiting the translocation of these assimilates to the outermost areas of leaves as a way to maintain the sucrose export and consequent cell integrity and functioning [20]. According to Karimi et al. [21] and Rehman et al. [22], the use of shading nets to increase the incidence of light in the red wavelength by making the plant’s photosynthetic apparatus more efficient results in more growth, corroborating the present study.

The higher stem elongation under the red-colored shade net and red light is related to the better relationship between red and far-red provided by both instruments for manipulating the light spectrum. Red-colored shade nets are characterized by having lower red to far-red and blue to far-red ratios relative to the natural radiation parameters; thus, the increased absorption of red light can cause an imbalance in the red to far-red ratio [23], causing fewer phytochromes in the active form (Prf) to migrate into the cell and degrade PIF proteins, which act by triggering a cascade of reactions in gene signaling related to elongation [24].

Furthermore, phytochromes are in an inactive form under a lower red to far-red ratio, and are thus not able to destroy PIFs. Gibberellins also act at a higher intensity under this low red to far-red ratio, degrading DELLA repressors, which, in smaller amounts, do not bind to PIFs, inactivating them, and leading to an accumulation of PIF proteins, which express genes linked to cell elongation [25].

Stem elongation also correlates with the presence of auxins, which are hormones conserved in cultures illuminated with red light and degraded in cultures maintained under blue light [26]. The red to far-red ratio acts on auxin concentration through the transcriptional induction of YUCCA genes (responsible for auxin synthesis) by PIF4, PIF5 and PIF7 proteins [27,28]. As seen earlier, the preservation of a lower red to far-red ratio maintains a higher concentration of PIFs in the nucleus. Thus, higher elongation is common under this condition, since the presence of auxins acts to maintain apical dominance, reducing lateral sprouting and the consequent partition of assimilates by plant growth regions [29].

Stem elongation under lighting was observed both under white-and black-colored shade nets, although it did not differ statistically from treatments of red light under red-colored shade nets. According to Henrique et al. [30], this occurs because white- and black-colored shade nets influence the irradiance but do not change the spectrum transmitted to the plants, reducing the amount of light that reaches the stems and making them more morphologically adapted, i.e., the stems elongate towards the light to make up for this light deficiency. Moreover, Canada goldenrod is considered a short-day plant. Thus, the extension of the light period, simulating long days, favors the vegetative development of the plant, leading to more elongated stems [15].

Similar results were found by Almeida et al. [31] and Oren-Shamir [32] in the cultivation of Japanese lisianthus (*Eustoma grandiflorum* Raf.) and pittosporum (*Pittosporum* sp.), respectively. The authors observed longer stems in plants grown under red-colored shade nets. A longer stem length was observed in cordyline plants under red- and white-colored shade nets. Silva et al. [33] found the same result for seedlings of *Physalis* species, with a higher balance in the red to far-red ratio provided by colored shade nets.

The smaller diameter of Canada goldenrod inflorescences, when grown under red light LEDs only when shaded by the black shade net, can be explained by the reduction in luminosity produced by shading associated with light quality. In this case, it was due to the lower incidence of light in the red range, which favors apical dominance and vertical elongation of the inflorescence because it is rich in red and far-red range wavelengths [29,34].

The flower stem is composed of a vegetative part at the base and a reproductive part at the apex; however, their elongation does not follow the same pattern. The longer length of stems under full sun and with the white- and red-colored shade net and full sun is due to the higher light availability at red and far-red wavelengths.

With plants under full sun, all available radiation reaches their surface, and they are able to use it at the red and far-red range. The red-colored shade net can modulate the spectrum; however, it reduces the light incidence by half, transmitting to plants a higher number of wavelengths in the red and far-red range, thus enhancing photosynthesis [30,35].

Similar behavior was observed under the white-colored shade net, which also reduces the incidence, but transmits white light to the plants, with similar amounts between red and far-red, and a predominance of the active form of phytochrome, thus accelerating growth [36]. On the contrary, the black-colored shade net only reduced the radiation that reached the plants, with no contribution to light quality, which may explain the lower inflorescence elongation under this condition. According to Silva et al. [33], elongation under black shading only occurs when there is an excess of shade, a situation in which the plant elongates as a means to overcome light deficiency, which was not observed in the present study.

The use of LED lights in cultivation also increased the inflorescence length, regardless of the emitted light spectrum. It occurs because Canada goldenrod plants are classified as short-day plants [15]. Thus, similar to that which occurs with the vegetative part of the stem, the lengthening of the lighting period influences the length of the internodes from which the florets are emitted.

Although the number of leaves is a highly responsive characteristic regarding light, different plant species can respond differently to changes in the light spectrum. In general, the use of shading and consequent decrease in luminosity leads to an increase in the number of leaves as a result of expanding the light-intercepting leaf area, which can be observed in begonia varieties, ornamental sunflowers (*Helianthus annuus* L.) and cordyline (*Cordyline terminalis* (L.) Kunth) [37,38,39], but was not verified in this study.

The increase in the number of leaves can also be a response to the higher availability of higher-quality light; that is, in the photosynthetically favorable range for producing photoassimilates, which provide higher growth when more readily available in the plant. This characteristic is usually provided by colored shade nets. Kong and Nemali [40] observed a higher number of leaves in lettuce (*Lactuca sativa* L.) plants grown under red light, which was also observed by Ilić et al. [41] in lettuce plants when grown under red- and pearl-colored shade nets. Silva et al. [14] also observed an increase in the number of leaves in heliconia grown under a red-colored shade net compared to cultivation under black- and blue-colored shade nets and full sun.

Other species seem to be less sensitive to changes in the spectrum provided by different-colored shade nets, with no changes in the number of leaves, as observed in studies of Canada goldenrod, jelly palm (*Butia capitata* (Mart.) Becc.) [42], and *Physalis* species [32]. In these cases, plants can respond to changes in luminosity not only by increasing the number of leaves but by other mechanisms, such as the expansion of the leaf blade and by increasing the thickness of tissues that capture the incident light, such as the palisade parenchyma, making them more efficient [43].

The lower fresh stem biomass of plants grown under black-colored shade nets is related to lower growth under this condition, as shown by the analysis of other variables, such as inflorescence length and scape diameter, in which stems had the lowest result, and also stem length, in which the treatment under black-colored shade net was associated with the absence of light, providing the shortest elongation (43.65 cm), negatively influencing the overall mean for this colored shade net.

Plant growth is defined as an increase in plant size, which is a function of biomass production driven by photosynthetic activity [44]. Therefore, the biomass response is a result of additional light energy provided for photosynthesis activity. According to [45], heliophytic plants efficiently use high radiation intensities due to the high capacity of their electron transport system, thus achieving higher photosynthetic gains and resulting in greater biomass.

Thus, there was a lower accumulation of fresh biomass due to a reduction in luminosity by 50%, due to zero transmittance to the plants of the incident light on the black-shade net. This reduction in brightness, without at least compensating for the light quality, resulted in a reduction in photosynthesis and formation of photoassimilates that could be made available for the growth and consequent accumulation of fresh mass by the plant. A similar situation was observed by [46] on basil plants grown under three shade nets and in full sun.

The absence of LED lighting, also associated with photosynthesis, reduced the biomass of the flower stems. However, in this case, the absence of light reduced the amount of time that the plant was exposed to light and, consequently, the synthesis of carbohydrates. In addition, as it is a short-day plant, the Canada goldenrod plant requires a greater supply of light for the stems to elongate, which, if not occurring, results in less fresh biomass. This need for the sufficient quality and quantity of light for ideal plant growth, resulting in greater stem biomass, was also described by Zeng and Labeke [11] in a study on the production of Chrysanthemums, where inadequate light spectra did not allow for adequate plant development, which resulted in lower biomass accumulation.

The smaller width of the base of the scape under black shade net may be related to an excessive growth of the stem towards the light, not being accompanied by an increase in the diameter, in order to maintain the quality of the stem. Although the white and red shade nets also have a 50% light-retention capacity, they are able to improve the range of the spectrum incident on the plant by increasing the input of diffused light, while the black shade nets behave in a neutral way [39,47,48].

Ludwig et al. [49] state that there must be a good relationship between plant height and stem diameter, as tall and thin stems tend to have reduced support, making them more susceptible to breakage, thus reducing the aesthetic quality or even making commercialization impossible. Furthermore, stems with a larger diameter correlate positively with the vase life of cut flowers because they are less prone to air embolism, caused by small-air bubbles-induced obstructions of vertical water flow into the stem, and because they allow for greater efficiency in the translocation of water and nutrients [50].

According to Silva et al. [33], the reduction in light intensity without an improvement in the quality of light offered, as is the case when shading with a black-shade net, can lead to the formation of etiolated plants of poor quality. According to the authors, an increase in plant length, not accompanied by an increase in stem thickness, may also indicate an imbalance in light.

Some authors have investigated the diameter of stems of flowers or plant stems in ornamental plants grown under different light spectra. In [51], it was found that the diameter of lisianthus stems was greater in plants grown without shading when compared to plants under a red shade net. For this same species, [31] found no difference in stems grown in full sun and under red, blue and black shade nets; the same result was found by [14] for heliconia stems, cv. ‘Golden Torch’.

The data show the need for light to be supplied to tango plants, in terms of both the photoperiod duration and light quality. This was evident when analyzing the number of stems emitted per plant and per square meter in the treatment without lighting and with the black shade net, compared to the other treatments. This treatment had the lowest production and productivity; however, it is clear that improvements in any one factor involved in the study, be it the duration, quantity or quality of light, provided an improvement in the results. Oren-Shamir et al. [32] state that plants are able to sense the quality, quantity, duration and direction of light and use it as a signal to optimize growth and development in a given environment.

Another example that can be observed is the greater production of stems in plants covered with red shade net which, despite the 50% reduction in light during the period of natural lighting, obtained gains with the extension of the photoperiod with the use of white light LEDs instead of red LED light. Mehraj et al. [52], studying flowering in three gerbera (*Gerbera jamesonii* Bolus and Hook) cultivars grown under seven light qualities, observed that, under a black shade net environment, there was a lower number of stems per plant (10.4 stems.plant^−1^).

After analyzing all the variables studied, it is possible perceive that the modulation of light quality during the cultivation of Canada goldenrod is capable of influencing both the production and the quality of stems of this species. In particular, considering the length and biomass, which are the main variables that contribute to an increase in the market value of Canada goldenrod floral stems, with the use of colored shade nets and LED lighting, all the treatments studied obtained the minimum values needed to meet the requirements of the Brazilian market, with a minimum weight of 10g and 40 cm in length per floral stem. However, the combination of white and black shade nets associated with the absence of LED lighting presented values very close to the minimum acceptable by the market in relation to length (43.7 and 48.7 cm), which may subject producers who adopt these practices to the risk of not providing a quality product or even not reaching the minimum standards in different harvests, with other variables influencing production.

The use of red or white LED lighting, together with white and black shade nets, allowed for the production of stem flowers over 70 cm long, while ed LED lighting under the red shade net had stem flowers averaging 81.4 cm. All the stem flowers with these treatments fell into higher ranges of the classification, and could be marketed as superior products with high added value.

In addition to the presented results, this study seeks to contribute to culture management and emphasize the importance of new research in the area for the identification of technologies and strategies that can be adopted by flower producers in order to obtain a higher-quality and profitable final product.

Based on this study and the proof of the beneficial effect of modulating light quality on the greater production and quality of Canada goldenrod floral stems, other more detailed studies can be carried out to increase the results, such as developing a greater understanding of the physiology of the plant in different light conditions, or the use of other forms, or even other sources, capable of modifying the incident light, such as lamps emitting other wavelengths, shade nets in other colors, and extensions of the lighting time, among others. It should also be noted that the response obtained is the result of the interaction of the plants with the environment in which they grow, and that the results obtained are not universal for the cultivation of Canada goldenrod, as different cultivation conditions, such as soil, climate and light throughout the year, can modify the responses, requiring detailed studies for each cultivation condition.

## 4. Materials and Methods

The experiment was conducted between August and December 2019, at the Experimental Farm belonging to “Universidade Estadual do Oeste do Paraná” (Unioeste), Marechal Cândido Rondon Campus, Paraná State, Brazil. The geographical coordinates of the experiment installation are 24°31′ S and 54°01′ W, at 420 m above the sea. The climate, according to Köppen, is Cfa type, mesothermic humid subtropical [53]; the predominant soil is Eutroferric RED LATOSOL [54]. Annual rainfall varies between 1600 and 1800 mm, with well-distributed rainfall throughout the year and hot summers. The average temperature of the region is between 22 and 23 °C and the relative humidity between 70 and 75% [55].

**Figure 4:**
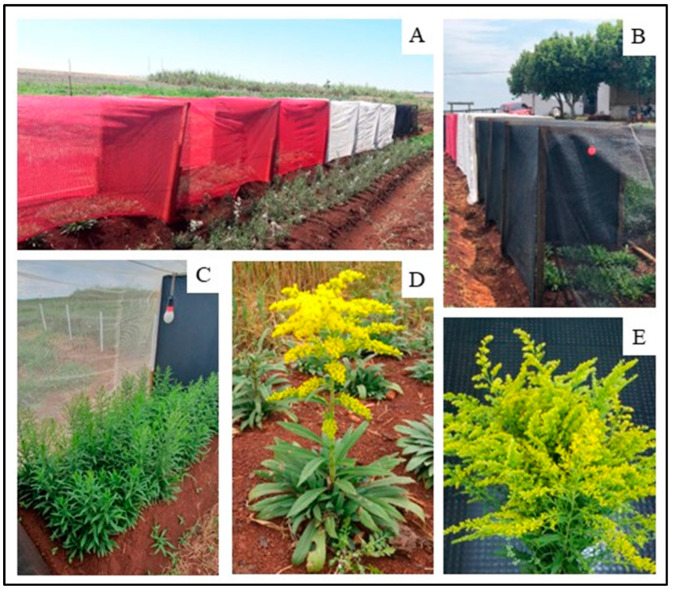
Side view of the experiment with colored shading nts and LED lights on Canada goldenrod (*Solidago canadensis* L.) plants (**A**,**B**); Canada goldenrod plants at the beginning of flowering (**C**); Canada goldenrod plant in full flowering (**D**); and stems flower collected for phytotechnical evaluation (**E**).

The ornamental species (*Solidago canadensis* L.) (Figure 4D) was obtained from cuttings taken from mother plants, kept in the greenhouse and rooted in protected cultivation containing washed sand of a medium texture. After three weeks, they were transplanted into plastic bags (8 × 10 cm) filled with a mixture of soil + manure, in a 1:1 (*v*/*v*) proportion, where they remained until the experiment was set up.

In the experimental area, a patch measuring 30 m × 1.0 m × 0.3 m (length, width and height, respectively) was raised and prepared, fertilized with 5 kg of manure, which was incorporated with a tractor. Next, the plants were planted, with a spacing of 0.20 × 0.20 m and arranged in four planting lines, following the statistical distribution planned for the experiment, with three experimental plots of 8 plants. After planting, the irrigation system was installed.

One week after planting, possible failures were repaired, and the lighting system and cover (shading) were installed. The lighting system was mounted with a continuous wire, suspended at a height of 1.50 m; all the nozzles were connected with the lamps, according to the distribution of the treatments. For every three experimental plots, corresponding to the repetitions of the same treatment, a rectangular wooden partition (1.20 m wide × 1.50 m high) was installed and was completely enclosed with black canvas in order to prevent light from entering the area. These same partitions served as support for the electric wire, to which the lamps were connected (Figure 4A,B).

The lamps used were LED, Superled model, from the Ourolux^®^ brand, with white and red light and had 9 W of power. According to the manufacturer, the white lamp has 810 lumens of luminous flux and a color rendering index (CRI) of 80, while the red lamp has 600 lumens and a CRI of 80. The lamps were installed 1.60 m apart, centered in the area to be illuminated, meeting the minimum requirement of 80 lux [56] (Figure 4C). The lighting wire was coupled to a timer and programmed to activate from 18:00 h to 22:00 h, extending the daily luminosity period to 16 h. In addition to the treatments with lamps, the experiment included treatments without the use of lighting. The lighting period was the same for all treatments, lasting 60 days from the installation of the lighting system, with a 15-day break and subsequent resumption of lighting until the time of harvesting the evaluated stems approximately 110 days after planting.

The cover of the plants was mounted with white, red and black shadings, with 50% light retention, and a height of 1.50 m (Figure 4A,B). The full sun treatment was not covered with any of the shading nets. Shading with shade nets was maintained from one week after planting the seedlings until the end of the experiment. Regarding the shade nets, all were from the Solpack^®^ brand. According to the manufacturer, all the nets have 50% shading and are made of Raschel mesh and special additives with high-density polyethylene, also containing UV treatment, which prevents the nets from drying out.

During the crop cycle, the cultural treatments inherent to the crop were carried out, such as irrigation and fertilization, according to recommendations in the literature and previous soil analysis. After three months, flowering began with elongation of the scape and emission of the flower stems (Figure 4C). When the flower stems were ready to be harvested (>40% open flowers), they were removed from plants to carry out the quality assessments (Figure 4E).

**Figure 5:**
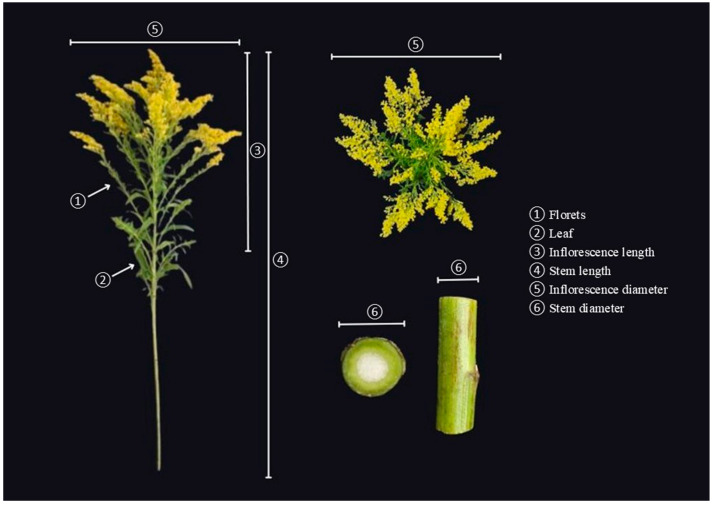
Phytotechnical evaluations performed on Canada goldenrod (*Solidago canadensis* L.) floral stems produced under different colored shade nets and LED lights.

The quality evaluations, or phytotechnics, performed were: stem length (cm) and inflorescence length (cm), measured by the distance between the insertion point of the most basal floret of the inflorescence and the stem tip with a graduated ruler; inflorescence diameter (cm), represented by the distance between the two ends of the basal ramets of the inflorescence and diameter of the stem base (floral scape) (mm), measured with a digital caliper (Stainless Hardened Digital brand); number of floral ramets/inflorescence, number of leaves (basal stems at the beginning of the ramets), determined from manual counting and; and fresh stem biomass (g), obtained with the aid of a digital analytical balance (Gehaka analytical balance AG200) (Figure 5). To determine these parameters, seven to eight inflorescences of each repetition were harvested, totaling 20 stems, divided into four repetitions of five stems; we attempted to collect at least one inflorescence per plant.

In addition to the quality parameters, the productive parameters were also evaluated, including the harvest and counting of all the floral stems produced in the experimental plot. Later, based on these data, the productivity was estimated, considering a planting density of 24 plants per square meter. All parameters described were analyzed for their importance in contributing to the quality of the floral stems, adding value to the product, as well as the productivity parameters established, because the quantity produced is of great economic relevance.

The experimental design used was randomized blocks, in a 4 × 3 factorial scheme [4 types of shading (black screen 50%, red screen 50%, white screen 50% and full sun) × 3 lighting conditions (red light, white light and no lighting)], containing 3 replications of 8 plants, totaling 12 treatments, 36 experimental plots, and 288 plants. The data collected at the end of the experiment were subjected to analysis of variance for homogeneity using the Bartlett test and normality using the Shapiro–Wilk (*p* > 0.05) test. Then, an analysis of variance was conducted by applying Tukey’s test, to compare means at a 5% error probability, using the SISVAR statistical program [57].

## 5. Conclusions

Canada goldenrod plants require an extension in the light period with artificial lighting to produce higher-quality stems.

The extension in the light period with artificial lighting can be conducted with red or white light bulbs, thus promoting the elongation and increase in the production of Canada goldenrod stems.

Red- and white-colored shade nets with 50% shading promote higher elongation and higher production of Canada goldenrod stems; however, they are not essential to obtain stems with the minimum standard acceptable by the Brazilian consumer market.

In addition to the scientific results, the study demonstrates an improvement in productivity and, above all, in the quality of the Canada goldenrod stems produced, which, in practice, represents greater added value to the product and thus represents an economic and social gain, given that the species is produced mainly on small rural properties.

## Figures and Tables

**Table 1:** Diameter of the floral scape of Canada goldenrod, cultivated under shade nets of different colors.

Shade Net Color	Flower Scape Diameter (mm)
Black	4.97 ± 0.82 b *
White	5.87 ± 0.54 ab
Red	5.31 ± 1.2 ab
Full sun	6.28 ± 1.24 a
CV (%)	19.85

* Means followed by the same letter in the column do not differ from each other according to Tukey’s test at a 5% error probability. CV = coefficient of variation.

**Table 2:** Number of stems per plant and number of stems per square meter in Canada goldenrod (*Solidago canadensis* L.) plants grown under lamps with different light qualities and shade nets of different colors.

Shade Net Color	Stem Flower Number.Plant^−1^		
	Red Light	White Light	Without Light
Black	2.5 ± 0.8 Aa *	2.6 ± 1.6 Ba	1.7 ± 0.9 Ba
White	2.1 ± 0.7 Aa	3.0 ± 0.1 ABa	3.2 ± 0.4 Aa
Red	3.0 ± 0.3 Aab	4.2 ± 0.4 Aa	1.9 ± 0.4 ABb
Full sun	1.8 ± 0.2 Aa	2.9 ± 0.4 ABa	2.0 ± 0.2 ABa
CV (%)	9.35		
Stem flower number.m^−2^			
	Red light	White light	Without light
Black	60.3 ± 19.55 Aa *	63.2 ± 28.87 Ba	41.0 ± 21.70 Ba
White	59.3 ± 17.02 Aa	72.0 ± 3 ABa	77.8 ± 10.29 Aa
Red	71.0 ± 7.55 Aab	100.3 ± 10.39 Aa	46.1 ± 8.79 ABb
Full sun	43.0 ± 4.58 Aa	68.0 ± 10.54 ABa	48.8 ± 4.17 ABa
CV (%)	11.69		

* Means followed by the same uppercase letter in the column and lowercase in the row do not differ from each other according to Tukey’s test at a 5% error probability. CV = coefficient of variation.

## Data Availability

The data supporting the findings of this study are not publicly available because there is no specific database for making the original research data available at the institutions that designed the study. For a better understanding of how the data were processed and interpreted, please request them from the corresponding author.
